# ISCB Public Policy Statement on Open Access to Scientific and Technical Research Literature

**DOI:** 10.1371/journal.pcbi.1002014

**Published:** 2011-02-24

**Authors:** Richard H. Lathrop, Burkhard Rost, ISCB Membership, ISCB Executive Committee, ISCB Board of Directors, ISCB Public Affairs Committee

## Preamble

The International Society for Computational Biology (ISCB) is dedicated to advancing human knowledge at the intersection of computation and life sciences. On behalf of the ISCB members, this public policy statement expresses strong support for open access, reuse, integration, and distillation of the publicly funded archival scientific and technical research literature, and for the infrastructure to achieve that goal.

Knowledge is the fruit of the research endeavor, and the archival scientific and technical research literature is its practical expression and means of communication. Shared knowledge multiplies in utility because every new scientific discovery is built upon previous scientific knowledge. Access to knowledge is access to the power to solve new problems and make informed decisions. Free, open, public, online access to the archival scientific and technical research literature will empower citizens and scientists to solve more problems and make better, more informed decisions. Attribution to the original authors will maintain consistency and accountability within the knowledge base. Computational reuse, integration, and distillation of that literature will produce new and as yet unforeseen knowledge.

We strongly encourage open software, data, and databases, issues that are not addressed here. A prior ISCB public policy statement on sharing software provides very clear support for open source/open access (http://www.iscb.org/iscb-policy-statements/software_sharing). We support open database access, standards, and interoperability. We also recognize that databases are complex dynamic entities, with ongoing roles and needs that cannot be treated properly within this statement. In contrast, the publicly funded archival research literature, addressed here, is the static historical record of publicly funded research outcomes.

ISCB supports many of the principles set forth in other open-access policies and statements, including the “Budapest Open Access Initiative,” the “Bethesda Declaration on Open Access Publishing,” the Bulletin of the World Health Organization “Equitable Access to Scientific and Technical Information for Health,” the US National Academies of Sciences report on “Sharing Publication-Related Data and Materials: Responsibilities of Authorship in the Life Sciences,” the Organisation for Economic Co-Operation and Development “Principles and Guidelines for Access to Research Data from Public Funding,” and the “Berlin Declaration on Open Access to Knowledge in the Sciences and Humanities.” Details on the documents mentioned here may be found in Text S1. Further background material is available in Text S2.

The public policy statement (Box 1) put forward here builds upon these principles to elucidate in more detail the public policy position of ISCB and its members on this important issue in scientific dissemination.

**Box 1. Public Policy Statement**

The International Society for Computational Biology strongly advocates free, open, public, online (i) access by person or machine to the publicly funded archival scientific and technical research literature; and (ii) computational reuse, integration, and distillation of that literature into higher-order knowledge elements.

## Supporting Statements

1. The possibilities latent in the digital information age make it essential to achieve open access, and computational reuse, integration, and distillation, of the publicly funded archival research literature.
  - Immediate access is preferable, and when access is at an interval following publication, that interval should not exceed one year.
  - At a minimum, every scientific journal should offer an open access option to every published research paper, as does every official or affiliated journal of the ISCB.
  - Copyright licenses explicitly should permit computational reuse, integration, and distillation, using standard existing language that eliminates the need for manual or legal review.
  - The format of the available article should be easy to parse by both human and machine (e.g., HTML). Ideally, a plain text version should be available as well (e.g., TXT), to facilitate computational reuse and integration (e.g., computational text mining for knowledge extraction).
  - Computational reuse, integration, and distillation should give attribution to the original authors.
2. Existing open access models show high impact, scientific benefit, feasibility, and acceptability.
  - The public benefit from open access to the world's online information via the publicly funded Internet provides a good model of expected impact.
  - The scientific fertilization from open access to genomic information via the publicly funded Human Genome Project provides a good model of expected scientific benefit.
  - Open access policies by the US National Institutes of Health, the Howard Hughes Medical Institute, and the Wellcome Trust provide good models of feasibility and acceptability.
  - The Creative Commons Attribution License and the Science Commons Open Access Data Mark provide good models of legal mechanisms for computational reuse, integration, and distillation.
3. Open literature access, reuse, integration, and distillation will enable a whole new generation of innovative computational tools and processes. The literature will be endowed with enriched commentary and usability. It will be connected seamlessly, by proper semantic links, to relevant Web sites, data, databases, and algorithms. Creating a web of knowledge around publications is an important consequence of semantic enrichment of the research literature. Such tools already are being built by publishers, researchers, entrepreneurs, and others. The further development of these tools should be supported aggressively. Removing all barriers to literature open access, reuse, integration, and distillation is critical to achieving such a knowledge-level transformation.
4. Supplementary data and methods should be openly available online, in sufficient detail to replicate the reported research results and facilitate reuse. Such material should be deposited in appropriate public repositories, in compliance with accepted community standards, and in accord with the existing ISCB public policy statement on sharing software. It should allow for application of other computational methods to the data and application of other data to the computational methods.
5. Publishing high-quality peer-reviewed scientific literature incurs costs. We recognize that cost recovery is a serious issue that must be addressed carefully if open access is to be a mandated policy.
6. Open-access policy details—which version, where stored, how annotated and organized, what incentives, etc.—must be considered carefully. However, it has now become essential to put forward a broad policy mandate for public access to, and computational reuse, integration, and distillation of, the publicly funded archival scientific and technical research literature.
7. This statement is intentionally neutral about any specific funding policy. Many implementations all may achieve the same essential goal. Acceptable funding policies should:
  - Remove barriers to open access and subsequent computational reuse, integration, and distillation.
  - Encourage public, private, and philanthropic funding organizations to establish policies that mandate free, open, public, online access to, and computational reuse, integration, and distillation of, the research results funded from their public, private, or philanthropic support.
  - Promote the body of publicly funded archival research literature as a public investment that bears interest, and not as an ongoing access cost to the public.
  - Establish copyright licenses in standard terms that permit literature access, reuse, and integration.
  - Specify a format that is easy to parse by both human and machine (e.g., HTML); and, ideally, also provide a plain text version (e.g., TXT) to facilitate computational reuse and integration.
  - Recognize the need to fund activities of peer review, copy editing, and publishing.
  - Provide fairness to several groups, including the developing world and its health concerns, unfunded or under-funded researchers, and others.
  - Provide fair interim support or compensation, if and where needed, to facilitate making transitions and adaptations to new models for publishing and sustaining essential revenue.
  - Be consistent with government laws, patent requirements, other existing regulations, and research dissemination through viable commercial mechanisms.
8. The expected cost of complete open access to the publicly funded archival research literature is only a very small percentage of the entire publicly funded international research endeavor. Nevertheless, it is undesirable to divert funding from current research and thus risk underfunding basic science. New funding should be made available for open-access policy implementations.

## Conclusion

Currently, scientific advancement is limited by article availability, access costs, copyright restrictions, document formats, bulk download limits, etc. All such barriers should be removed.

The publicly funded archival scientific and technical research literature represents a substantial investment by the public, governments, foundations, non-profit institutions, publishers, individuals, and others. We in the ISCB are committed to the continuous enhancement and leveraging of society's knowledge resources. One of our primary missions is the computational integration of individual pieces of knowledge from the research literature and databases, in ways that provide powerful new ideas and insights for next-stage research, for the benefit of the scientific community and society in general.

To achieve these public benefits, we strongly advocate free, open, public, online access to the publicly funded archival scientific and technical research literature, and the computational reuse, integration, and distillation of that literature into higher-order knowledge elements.

The example scenario shown in Box 2 illustrates an important public health benefit that could be achieved immediately: the opportunity to pursue useful knowledge-based innovations, by computational reuse, integration, and distillation of the publicly funded archival research literature, across many areas in biology and medicine.

**Box 2. Example Scenario**

An automated malaria Web site might access location-specific information from thousands of publicly funded malaria research articles daily, and then integrate that information into a free online interactive world map. Such a map might be annotated with up-to-date information about disease occurrences, drug resistance profiles, current best control practices, etc., as distilled from the research literature extracted for and attached to each local region. A hypothetical user might be a public health official in the developing world responsible for controlling a sudden malaria outbreak in a remote area. Such a Web site should encounter no barriers while performing this free, useful, and potentially essential public service.

*Example Discussion*

A search for “malaria” in the US NIH/NLM PubMed literature database yielded more than 55,000 hits (July 2010; http://www.ncbi.nlm.nih.gov/pubmed/). The publicly funded portion of these 55,000 “malaria” hits should be freely available in bulk to this hypothetical malaria Web site, using technologies well suited to bulk tasks, for purposes of (i) the initial bulk literature download; (ii) regular updates; and (iii) intermittent bulk repeat downloads to reinitialize an improved knowledge base. The relevant copyright permissions should permit computationally recombining the publicly funded portion of these 55,000 texts into whatever final form is most useful and informative to the user.

## Supporting Information

Text S1 Documents Mentioned in the Statement Text
(DOC)

Text S2 General Background Material, Other Statements, and Materials
(DOC)

