# Supplementary material for: ISCB Public Policy Statement on Open Access to Scientific and Technical Research Literature
Source: PLoS Comput Biol. 2011 Feb 24;7(2):e1002014. doi: 10.1371/journal.pcbi.1002014 (PMC3044765; doi:10.1371/journal.pcbi.1002014)
Supplement: Text S1 — Documents Mentioned in the Statement Text (DOC) [file pcbi.1002014.s001.doc]

**Text S1. Documents Mentioned in the Statement Text[[1]](#footnote-2)**

*1. Text of ISCB public policy statement on sharing software*

http://www.iscb.org/iscb-policy-statements-/187

*2. Text of the “Budapest Open Access Initiative.”*

http://www.soros.org/openaccess/read.shtml

http://en.wikipedia.org/wiki/Budapest_Open_Access_Initiative

*3. Text of the “Bethesda Declaration on Open Access Publishing.”*

http://www.earlham.edu/~peters/fos/bethesda.htm

*4. Text of the Bulletin of the World Health Organization “Equitable Access to Scientific and Technical Information for Health.”*

http://www.scielosp.org/scielo.php?script=sci_arttext&pid=S0042-96862003001000003

*5. Text of the U.S. National Academies of Sciences report on “Sharing Publication-Related Data and Materials: Responsibilities of Authorship in the Life Sciences.”*

http://www.nap.edu/openbook.php?isbn=0309088593

*6. Text of the OECD (Organisation for Economic Co-Operation and Development) “Principles and Guidelines for Access to Research Data from Public Funding.”*

http://www.oecd.org/dataoecd/9/61/38500813.pdf

*7. Text of the “Berlin Declaration on Open Access to Knowledge in the Sciences and Humanities.”*

http://www.eprints.org/events/berlin3/outcomes.html

http://oa.mpg.de/openaccess-berlin/berlindeclaration.html

http://en.wikipedia.org/wiki/Berlin_Declaration_on_Open_Access_to_Knowledge_in_the_Sciences_and_Humanities/

*8. Human Genome Project.*

http://genome.gov/

http://genome.energy.gov/

*9. Text of Open Access Policy from the U.S. National Institutes of Health.*

http://publicaccess.nih.gov/policy.htm

http://publicaccess.nih.gov/

*10. Text of Open Access Policy from the Howard Hughes Medical Institute.*

http://www.hhmi.org/about/research/sc320.pdf

http://www.hhmi.org/about/research/QA_papp.pdf

*11. Text of the Wellcome Trust's “Position Statement in Support of Open and Unrestricted Access to Published Research.”*

http://www.wellcome.ac.uk/About-us/Policy/Spotlight-issues/Open-access/Policy/index.htm

*12. Text of the Creative Commons Attribution License.*

http://creativecommons.org/licenses/by/3.0/

*13. Text of the Science Commons Open Access Data Mark.*

http://www.sciencecommons.org/projects/publishing/open-access-data-protocol/

1. These URLs were correct when this statement was written, but are by their nature ephemeral and not archival. [↑](#footnote-ref-2)
