# Supplementary material for: ISCB Public Policy Statement on Open Access to Scientific and Technical Research Literature
Source: PLoS Comput Biol. 2011 Feb 24;7(2):e1002014. doi: 10.1371/journal.pcbi.1002014 (PMC3044765; doi:10.1371/journal.pcbi.1002014)
Supplement: Text S2 — General Background Material, Other Statements, and Materials (DOC) [file pcbi.1002014.s002.doc]

**Text S2. General Background Material, Other Statements and Materials[[1]](#footnote-2)**

*1. Academic publishing — Wikipedia, the free encyclopedia.*

http://en.wikipedia.org/wiki/Academic_publishing

*2. Open access (publishing) — Wikipedia, the free encyclopedia.*

http://en.wikipedia.org/wiki/Open_access_(publishing)

*3. ROARMAP (Registry of Open Access Repository Material Archiving Policies) as recommended by the Berlin Declaration.*

http://www.eprints.org/openaccess/policysignup/

*4. Text of Public Library of Science “Open Letter to Scientific Publishers” (signed by ~34,000 scholars worldwide).*

http://www.plos.org/about/letter.php

*5. Text of Research Councils of the UK “Access to Research Outputs.”*

http://www.rcuk.ac.uk/cmsweb/downloads/rcuk/documents/2006statement.pdf

http://www.rcuk.ac.uk/cmsweb/downloads/rcuk/news/oareport.pdf

http://www.rcuk.ac.uk/research/outputs/default.htm

*6. Text of European Research Advisory Board Final Report “Scientific Publication: Policy On Open Access.”*

http://ec.europa.eu/research/eurab/pdf/eurab_scipub_report_recomm_dec06_en.pdf

*7. Open Science Directory.*

http://www.opensciencedirectory.net/

*8. Peter Suber's “Open Access Overview.”*

http://www.earlham.edu/~peters/fos/overview.htm

http://www.earlham.edu/~peters/fos/brief.htm

*8. Scholarly Kitchen on the Open Access Financial Model*

http://scholarlykitchen.sspnet.org/2010/09/14/why-the-open-access-financial-model-will-continue-to-transmogrify/

1. These URLs were correct when this statement was written, but are by their nature ephemeral and not archival. [↑](#footnote-ref-2)
